# NMDARs antagonist MK801 suppresses LPS-induced apoptosis and mitochondrial dysfunction by regulating subunits of NMDARs via the CaM/CaMKII/ERK pathway

**DOI:** 10.1038/s41420-023-01362-9

**Published:** 2023-02-11

**Authors:** Wei-Min Han, Xiao-Bin Hao, Yi-Xiang Hong, Shan-Shan Zhao, Xu-Chang Chen, Ruiying Wang, Yan Wang, Gang Li

**Affiliations:** 1 Xiamen Cardiovascular Hospital of Xiamen University, School of Medicine, Xiamen University, Xiamen, Fujian 361000 China; 2 The First Affiliated Hospital of Soochow University, Medical College of Soochow University, Suzhou, China

**Keywords:** Apoptosis, Receptor pharmacology, Drug regulation

## Abstract

Lipopolysaccharide (LPS) displays a robust immunostimulatory ability upon Toll-like receptor 4 (TLR4) recognition. N-methyl-D-aspartate receptors (NMDARs) are highly compartmentalized in most cells and implicated in various inflammatory disorders. However, the relationship between TLR4 and NMDARs has not been explored deeply. This study aimed to examine the role of NMDARs and its specific inhibitor MK801 in LPS-treated endothelial cell dysfunction and the related mechanism in vivo and in vitro. The results showed that pre-treatment with MK801 significantly decreased LPS-induced cell death, cellular Ca^2+^, cellular reactive oxygen species, and glutamate efflux. Moreover, MK801 restrained LPS-induced mitochondrial dysfunction by regulating mitochondrial membrane potential and mitochondrial Ca^2+^ uptake. The oxygen consumption, basal and maximal respiration rate, and ATP production in LPS-treated HUVECs were reversed by MK801 via regulating ATP synthesis-related protein SDHB2, MTCO1, and ATP5A. The molecular pathway involved in MK801-regulated LPS injury was mediated by phosphorylation of CaMKII and ERK and the expression of MCU, MCUR1, and TLR4. LPS-decreased permeability in HUVECs was improved by MK801 via the Erk/ZO-1/occluding/Cx43 axis. Co-immunoprecipitation assay and western blotting showed three subtypes of NMDARs, NMDAζ1, NMDAε2, and NMDAε4 were bound explicitly to TLR4, suppressed by LPS, and promoted by MK801. Deficiency of NMDAζ1, NMDAε2, or NMDAε4 induced cell apoptosis, Ca^2+^ uptake, ROS production, and decreased basal and maximal respiration rate, and ATP production, suggesting that NMDARs integrity is vital for cell and mitochondrial function. In vivo investigation showed MK801 improved impairment of vascular permeability, especially in the lung and mesentery in LPS-injured mice. Our study displayed a novel mechanism and utilization of MK801 in LPS-induced ECs injury and permeability.

## Introduction

Gram-negative bacteria are related to many diseases or pathological states, including post-cardiac surgery, respiratory and urogenital tract infections, endocarditis, and gastritis [1, 2]. The crucial virulent factor of Gram-negative bacteria that induces the innate immune response or inflammation is lipopolysaccharide (LPS) [3]. LPS consists of a glycolipid terminal structure called the lipid A-core, responsible for the endogenous toxicity of LPS, and an O-antigen polysaccharide [4]. LPS activates the systemic and cellular inflammatory response mainly through Toll-like receptor 4 (TLR4), expressed in almost all cell types [5]. LPS also induces local inflammatory pathologies, mitochondrial dysfunction, and imbalanced generation of reactive oxygen species (ROS) [6, 7]. The experimental report has shown that LPS-induced inflammation depends on ROS and the associated downstream MAPK signaling pathways, including ERK, JNK, and p-38 [8]. LPS-regulated Ca^2+^ release from ER stores underlies nuclear factor-kappa B activation and downstream inflammatory signaling in lung micro-vessels [9].

Excess glutamate release is a significant mechanism that leads to excitatory toxicity injury in various cells. The N-methyl-D-aspartate receptors (NMDAs) can bind to glutamate and are highly permeable to Ca^2+^, indicating a crucial role in excitatory toxicity disorders [10]. Despite different pathologies in various disorders, evidences suggest that NMDARs dysfunction is one of the common causes [11, 12]. Given the critical role of NMDARs in excitotoxicity, the initial treatment approach was to suppress the receptors with inhibitors [13]. A previous study showed that glial-localized NMDARs likely play a limited role in oligodendrocyte demise associated with tumor necrosis factor-α (TNF-α)- or LPS-induced chronic inflammation [14]. However, an NMDAR antagonist, memantine, was reported to attenuate bleomycin-induced acute lung injury [15], and another NMDAR antagonist, quinolinic acid, can relieve the development of LPS-induced depressive-like behavior in C57BL/6J mice [16], indicating that NMDARs may be related to LPS-induced systemic inflammatory response or other dysfunctions. Our present study was designed to profoundly investigate the role of NMDARs antagonist MK801 on LPS-induced human umbilical vein endothelial cells (HUVECs) damage, such as Ca^2+^ uptake, ROS production, mitochondria function, and cell permeability in vitro and LPS-induced acute lung injury in vivo, as well as the potential mechanism involved in these processes.

## Results

### MK801 improved the LPS-induced decrease of cell viability in HUVECs

To investigate the functional impact of NMDARs inhibitor MK801 on HUVEC, we examined the cell viability in different concentrations of MK801 (1, 5, and 10 μM) treated HUVECs in the absence or presence of LPS.

**Fig. 1:**
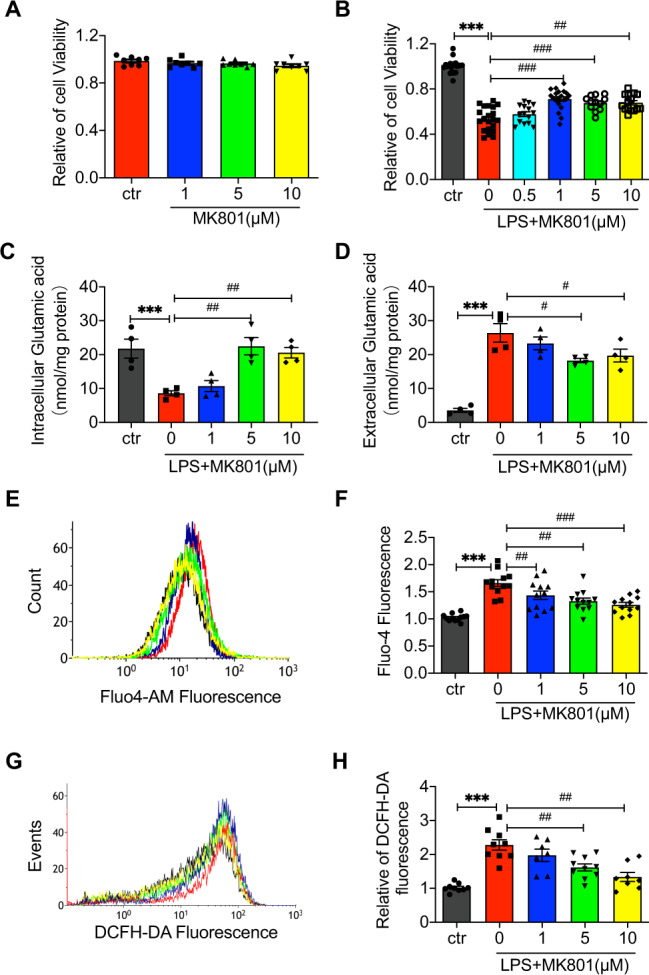
MK801 protects against LPS-treated HUVECs. **A** MTT assay showed MK801 (1, 5, 10 μM) did not affect cell viability in HUVECs. **B** MTT assay showed MK801 (1, 5, 10 μM) effectively countered viability reduction induced by LPS (20 μg/ml). **C** Intracellular glutamate and **D** extracellular glutamate concentrations in HUVECs after treatment with LPS (20 μg/ml) for 24 h in the absence or presence of MK801(1, 5, 10 μM). **E** Flow cytometry graphs and **F** analysis of Ca^2+^ influx in HUVECs with the fluorescent probe Fluo-4-AM in response to LPS (20 μg/ml) and MK801 (1, 5, 10 μM). **G** Flow cytometry graphs and (H) analysis of ROS production in HUVECs treated with LPS (20 μg/mL) in the absence or presence of MK801 (1, 5, 10 μM) for 24 h. (Data are presented as mean ± SEM, ***P < 0.001, **P < 0.01, *P < 0.05 vs. Control; ###*P* < 0.001, ##*P* < 0.01, #*P* < 0.05 vs. LPS).

The results showed that different concentrations of MK801 did not influence cell viability (Fig. 1A). Moreover, pretreatment with MK801 ameliorated LPS-decreased cell viability (Fig. 1B).

The potential mechanism of MK801 on LPS-induced cell injury was determined in the concentration of intracellular and extracellular glutamic acid, intracellular Ca^2+^, and ROS production. The intracellular glutamic acid was significantly decreased (Fig. 1C), and extracellular glutamate was increased in LPS-exposed HUVECs (Fig. 1D), while the NMDARs inhibitor can dramatically reverse these alterations induced by LPS. The cellular Ca^2+^ and ROS in HUVECs were also elevated by LPS; pretreatment of MK801 significantly restrained the elevation of cellular Ca^2+^ (Fig. 1E, F) and ROS (Fig. 1G, H). These results indicated that LPS-induced cell injury was promoted by cellular glutamate release, Ca^2+^ uptake, and ROS production, which can be reversed by NMDARs inhibitor MK801.

### MK801 reversed LPS-induced dysfunction of mitochondria in HUVECs

**Fig. 2:**
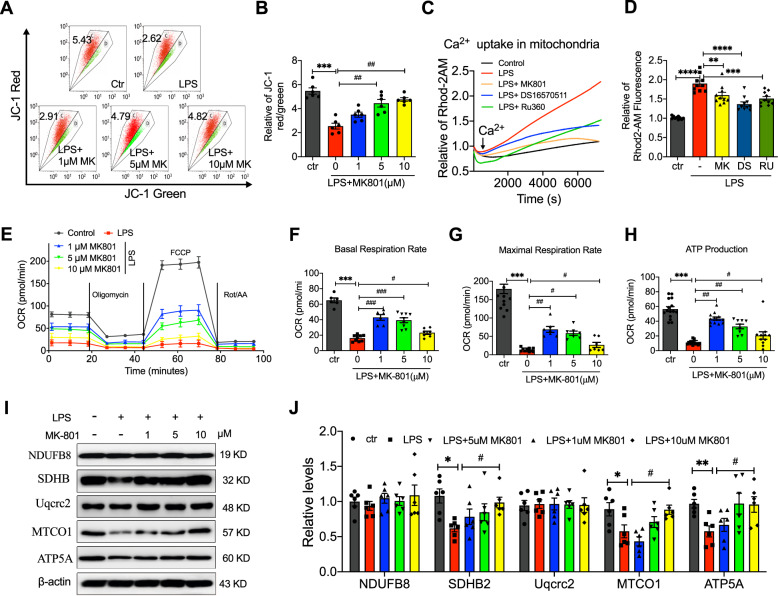
Protective effects of MK801 against LPS-induced mitochondrial dysfunction in HUVECs. **A** Flow cytometry graphs and **B** analysis of mitochondrial transmembrane potential in HUVECs treated with LPS in the absence or presence of indicated doses of MK801. Representative of traces (**C**) and quantification at 10 min (**D**) of mitochondria Ca^2+^ influx labeled with Rhod-2 AM in HUVECs treated with LPS in the presence or absence of Ru360 (mitochondrial calcium uptake inhibitor), DS16570511 (MCU inhibitor), or MK801 for 2 h. **E**–**H** O_2_ consumption rates (**D**), basal (**E**) and maximal respiration rate (**F**), and ATP production (**G**) in HUVECs treated with LPS in the presence or absence of MK801. Representative of western blotting images (**I**) and quantification (**J**) of NDUFB8, SDHB2, UQCRC2, MTCO1, and ATP5a in HUVECs treated with LPS in the presence or absence of MK-801 at indicated doses. (Data are presented as mean ± SEM, ***P < 0.001, **P < 0.01, *P < 0.05 vs. Control; ###*P* < 0.001, ##*P* < 0.01, #*P* < 0.05 vs. LPS).

Abnormal mitochondrial energy metabolism and cell respiratory function are closely associated with mitochondrial dysfunction. The ROS production in Fig. 1 was decreased by MK801, indicating that MK801 may participate in LPS-induced cell injury by regulating mitochondria function. Therefore, we further explored the effect of MK801 on the potential of mitochondria and mitochondrial Ca^2+^ uptake using a mitochondrial potential indicator JC-1 and a mitochondrial Ca^2+^ indicator Rhod-2 AM, respectively. The potential of mitochondria (JC-1 red/green ratio, Fig. 2A, B) was significantly decreased by LPS, which can be substantially reversed by treatment of MK801. To further evaluate the mechanism of LPS- and MK801-regulated mitochondrial calcium uptake, a mitochondrial calcium uniporter (MCU) inhibitor DS16570511 and a mitochondrial calcium uptake inhibitor Ru360 were employed. Interestingly, LPS-stimulated Ca^2+^ uptake in mitochondria also is suppressed by DS16570511 and Ru360, indicating MCU is involved in LPS-regulated mitochondrial calcium, and MK801 can restrain it (Fig. 2C, D).

As an indicator of oxidative phosphorylation (OXPHOS) in mitochondrial respiration, oxygen consumption rate (OCR) was measured using an Agilent seahorse analyzer to validate the role of MK801 in mitochondrial energy metabolism. As illustrated in Fig. 2E–H, LPS-exposed HUVECs had the lowest resting basal respiration rate, maximal mitochondrial capacity, and ATP production, different concentrations of MK801-pretreated cells significantly promoted them, suggesting that MK801 protected against LPS-exposed HUVECs by promoting OXPHOS and ATP content.

To further investigate the pathway of MK801 regulated the OXPHOS in LPS-exposed HUVECs, we then detected the mitochondrial respiratory chain complexes (I, II, III, IV, and V), including NDUFB8, SDHB, Uqcrc2, MTCO1 SDHB2, and ATP5A. The results exhibited that the expression of SDHB2, MTCO1, and ATP5a, but not NDUFB8 and Uqcrc2, were significantly suppressed by LPS, and MK801 effectively restrained the decrease of SDHB2, MTCO1, and ATP5a, indicating that SDHB2, MTCO1, and ATP5a participated in MK801-regulated protection of mitochondrial respiration in LPS-induced HUVECs (Fig. 2I, J). Thus far, we have provided evidence that NMDAR inhibitor MK801 regulated LPS-induced mitochondria dysfunction and shifted cellular respiration in favor of OXPHOS by regulating expression SDHB2, MTCO1, and ATP5a.

### CaM and CaMKII participated in MK801-regulated LPS-induced cell injury

**Fig. 3:**
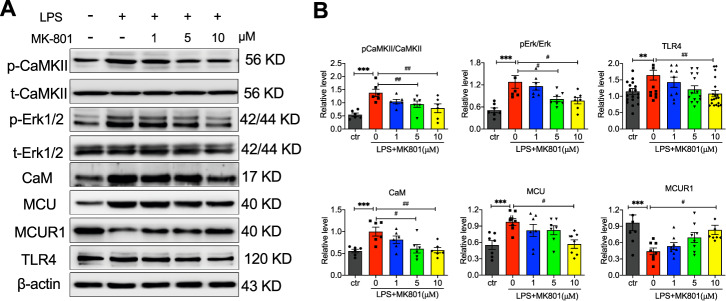
Molecular signal pathway in MK801 regulated dysfunction of HUVEC induced by LPS. **A** Representative of western blotting images and **B** analysis of phosphorylation of Erk1/2 (p-Erk1/2) and CaMKII (p-CaMKII), and expression of CaM, MCU, MCUR, and TLR4 in HUVECs exposed to LPS in the presence or absence of different concentrations of MK801. (Data are presented as mean ± SEM, ***P < 0.001, **P < 0.01, *P < 0.05 vs. Control; ###*P* < 0.001, ##*P* < 0.01, #*P* < 0.05 vs. LPS).

Previous evidence has demonstrated the binding of calmodulin (CaM) to NMDARs is required for CaM-dependent inactivation of NMDAR, and NMDARs-mediated CaMKII/ERK activation contributes to renal fibrosis [17, 18], indicated CAM, CAMKII, and ERK pathways are essential for NMDARs regulated cell function. To determine whether CAM, CAMKII, and ERK are involved in the effect of MK801 on LPS-induced cell injury and mitochondrial dysfunction, we examined the expression of CaM, CaMKII, Erk, MCU, a mitochondrial inner membrane calcium uniporter, and its receptor MCUR1, as well as TLR4. The results showed that MK801 decreased the LPS-induced phosphorylation of CaMKII and ERK, the expression of CaM, MCU, and TLR4, and promoted the expression of MCUR1 (Fig. 3A, B). These results indicated that CaM and CaMKII are involved in NMDAR inhibitor-regulated LPS-induced cell injury and mitochondrial dysfunction by regulating phosphorylation of ERK and MCU/MCUR1 pathway.

### MK801 restrained LPS-induced cell integrity and permeability by regulating the expression of ZO-1, Occludin, and Cx43 via the ERK pathway

**Fig. 4:**
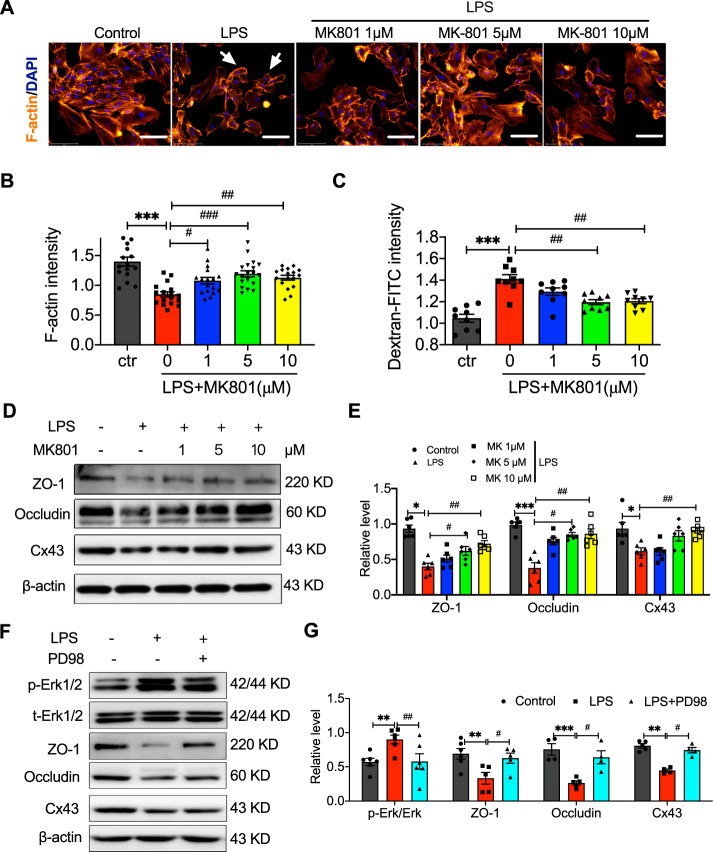
MK801 rescued LPS-increased permeability in HUVECs. **A** Immunostaining images of F-actin labeled with TRITC Phalloidin and **B** analysis in HUVECs induced by LPS and treated with MK801; the peripheral bands of F-actin and intercellular gaps appeared between adjacent cells. **C** Vascular Permeability Assay showed the relative intensity of FITC-Dextran in HUVECs induced by LPS and treated with MK801. Representative of western blotting images (**D**) and analysis (**E**) of expression of ZO-1, Occludin, and Cx43 in LPS-exposed HUVECs in the presence or absence of different concentrations of MK801. (Data are presented as mean ± SEM, ***P < 0.001, **P < 0.01, *P < 0.05 vs. Control; ###*P* < 0.001, ##*P* < 0.01, #*P* < 0.05 vs. LPS).

Epithelial and/or endothelial barriers play an important role in animals, including humans, and the tight junction is an essential component of these barriers [19]. Previous evidence has demonstrated that LPS can significantly damage the cell membrane integrity and permeability [20]. In our present investigation, we observed the role of MK801 in LPS-induced cell insults on the cell structure by using immunostaining with filamentous actin (F-actin), a critical cytoskeletal component protein, and FITC-conjunct Dextran, which can reflect the permeability of cells. The results showed that LPS significantly altered the cell membrane integrity (Fig. 4A), leading to curled up and shrinkage. The F-actin intensity decreasing in Fig. 4B indicated membrane integrity was reduced in LPS-exposed cells, which can be counteracted by pre-treatment with MK801. In addition, the cell permeability was examined by FITC-conjunct dextran. The relative intensity of dextran was elevated by exposure to LPS. MK801, especially at 5 or 10 μM, significantly restrained the elevation of permeability induced by LPS in HUVECs (Fig. 4C).

ZO-1, Occludin, and Cx43 are the critical regulators of cell integrity and permeability in cell-cell junctions [19, 21, 22]. We then observed the expression of ZO-1, Occludin, and Cx43 in LPS-treated cells in the presence or absence of MK801. We found that LPS significantly decreased the expression of ZO-1, Occludin, and Cx43, and MK801 can effectively prevent the decline of ZO-1, Occludin, and Cx43 (Fig. 4D, E). These data suggested that MK801-altered cell integrity and permeability in ECs were mediated by ZO-1, occluding, and Cx43.

Our previous results have observed the phosphorylation Erk1/2 has participated in MK801-regulated LPS-induced cell injury. We then employed PD98059, an antagonist of the Erk1/2 pathway, and found that PD98059 can significantly decrease phosphorylation of Erk1/2 and promote the decrease of ZO-1, Occludin, and Cx43 induced by LPS, indicating that the LPS-altered cell integrity was mainly regulated by Erk pathway (Fig. 4F, G).

### MK801 mediated LPS-induced injury by interacting and promoting NMDAζ1, NMDAε2, and NMDAε4

**Fig. 5:**
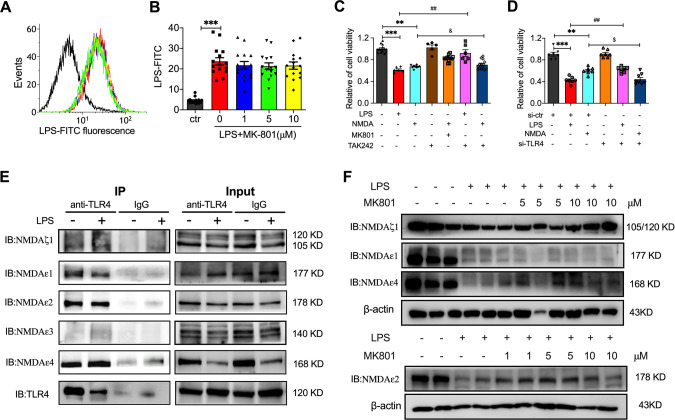
The relationship between TLR4 and NMDARs. **A** Flow cytometry images and **B** analysis of FITC-conjugated LPS-treated HUVECs in the presence or absence of indicated doses of MK-801. **C** MTT assay showed relative cell viability in NMDA- or LPS-treated HUVECs in the presence or absence of MK801 or TAK242. **D** MTT assay showed relative cell viability in control siRNA or TLR4 siRNA transfected HUVECs treated with LPS or MK801. **E** Co-IP detection showed the interaction of NMDARs subunits, NMDAζ1, NMDAε1, NMDAε2, NMDAε3, and NMDAε4, with TLR4 induced by LPS in HUVECs. **F** The western blotting image showed the expression of NMDAζ1, NMDAε1, NMDAε2, and NMDAε4 treated with LPS in the presence or absence of different concentrations of MK801 in HUVECs. (Data are presented as mean ± SEM, ***P < 0.001, **P < 0.01 vs. Control or control siRNA; ##*P* < 0.01 vs. LPS or control siRNA + LPS; &*P* < 0.05 vs. NMDA; $*P* < 0.05 *vs*. TLR4 siRNA).

To explore the relationship between the NMDAR inhibitor MK801 and LPS, the FITC-conjunct LPS was applied to determine whether MK801 regulated LPS-induced endothelial injury by affecting the interaction between LPS and MK801. Flow cytometry detection showed that the fluorescence of FIPC-conjunct LPS was not decreased by different concentrations of MK801 (Fig. 5A, B), which indicates that MK801 did not exert its role by directly interacting with LPS.

In order to inspect the downstream of NMDAR-regulated cell viability, we employed NMDA and TAK242, a selective TLR4 inhibitor, and TLR siRNA to explain the connection among NMDA, NMDARs, and TLR4. The results showed that, similar to LPS, NMDA significantly repressed cell viability; MK801 could substantially restrain NMDA’s effect; TAK242 can also suppress the effect of LPS and NMDA (Fig. 5C); which were validated by the knockdown of TLR4 (Fig. 5D). The above results implied that NMDA receptors regulated LPS-induced endothelial cell injury via the TLR4 pathway.

NMDARs are ionotropic channels formed by the combination of different subunits, including NMDAζ1 and four members of the NMDA2 family (NMDAε1, NMDAε2, NMDAε3, and NMDAε4) [23]. Consequently, co-immunoprecipitation staining was employed to further investigate whether MK801 exerted its function by regulating the interaction of TLR4 with the subunits of NMDARs, using antibodies against TLR4 and detected NMDAζ1, NMDAε1, NMDAε2, NMDAε3, and NMDAε4 in the precipitate. The results showed that TLR4 was explicitly connected with NMDAζ1, NMDAε1, NMDAε2, and NMDAε4, but not NMDAε3 (Fig. 5E).

Previous literature has documented that the administration of MK801 can significantly promote the expression of NMDARs in the rat prefrontal cortex in vivo [24]. However, whether LPS and MK801 regulated the expression of NMDAR subunits was not illustrated. Hence, we explored the expression of NMDAζ1, NMDAε1, NMDAε2, and NMDAε4, using western blot in LPS or/and MK801-treated HUVECs. The results showed that LPS treatment ECs significantly decreased the expression of NMDAζ1, NMDAε1, NMDAε2, and NMDAε4, and MK801 significantly elevated the expression of NMDAζ1, NMDAε2, and NMDAε4 (Fig. 5F). These results imply that MK801 exerts its role by promoting the expression of NMDAζ1, NMDAε2, and NMDAε4, which directly interact with TLR4.

### Knockdown of NMDAζ1, NMDAε2, and NMDAε4 induced HUVECs apoptosis, Ca^2+^ uptake, ROS production, and mitochondrial respiration dysfunction

**Fig. 6:**
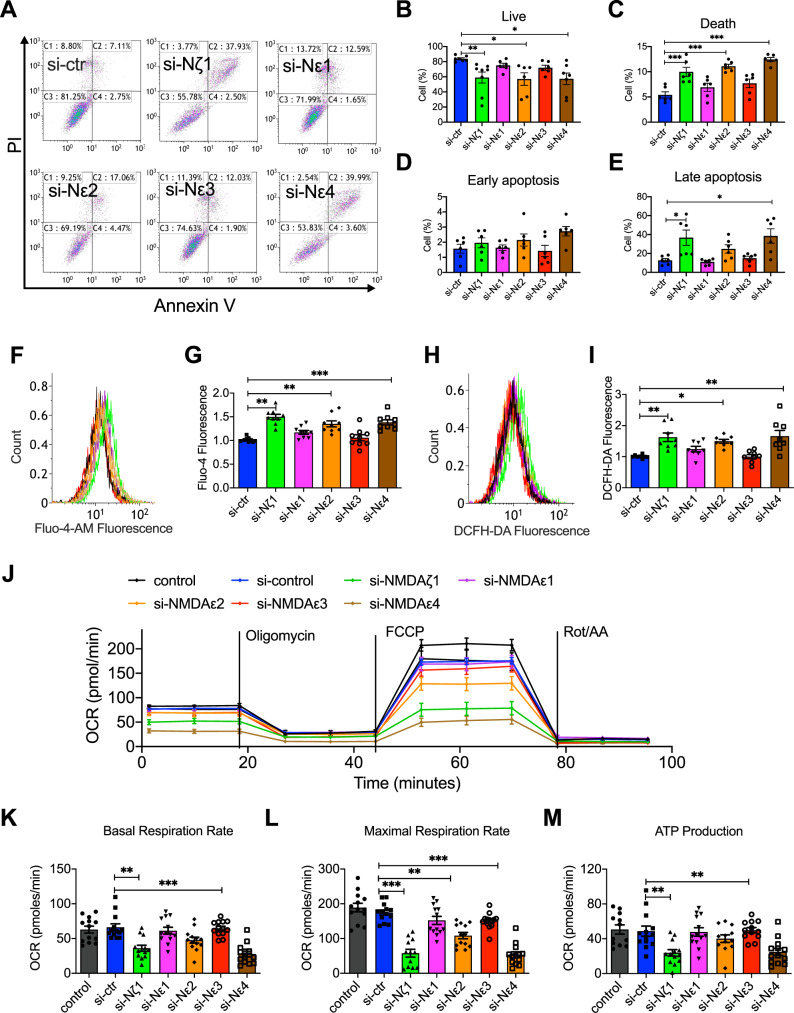
Knockdown of NMDAR subunits NMDAζ1, NMDAε2, and NMDAε4 promoted apoptosis, intracellular Ca^2+^ influx, ROS production, and restrained mitochondrial O_2_ consumption rate. **A** Representative flow cytometry images and analysis of cell apoptosis with live cells (**B**), death (**C**), early apoptosis (**D**), and late apoptosis in HUVECs transfected with control siRNA, NMDAζ1 siRNA, NMDAε1 siRNA, NMDAε2 siRNA, NMDAε3 siRNA, or NMDAε4 siRNA. **F** Flow cytometry graphs and **G** analysis of Ca^2+^ influx in HUVECs transfected with control siRNA, NMDAζ1 siRNA, NMDAε1 siRNA, NMDAε2 siRNA, NMDAε3 siRNA, or NMDAε4 siRNA. **H** Flow cytometry graphs and **I** analysis of ROS in HUVECs transfected with control siRNA, NMDAζ1, NMDAε1 siRNA, NMDAε2 siRNA, NMDAε3 siRNA, or NMDAε4 siRNA. **J** Seahorse XF24 Extracellular Flux Analyzer detected mitochondrial O_2_ consumption rate, **K** basal respiration rate, **L** maximal respiration rate, and **M** ATP production in HUVECs after transfection with different siRNA. (Data are presented as mean ± SEM, ***P < 0.001, **P < 0.01, *P < 0.05 vs. control siRNA).

Our previous results have shown that MK801 reversed the LPS-decreased expression of NMDAζ1, NMDAε2, and NMDAε4. To determine whether restraining the expression NMDARs exerts a similar role as the effect of LPS, we explored the apoptosis, Ca^2+^ uptake, ROS production, and mitochondrial respiration when knockdown of the NMDARs. Interestingly, in flow cytometry for apoptosis detection, the live cells (Fig. 6A, B) significantly decreased, and death (Fig. 6A, C) and late apoptotic cells (Fig. 6A, E) were increased after knockdown of NMDAζ1, NMDAε2, and NMDAε4, but not NMDAε1 and NMDAε3. Moreover, the Ca^2+^ uptake (Fig. 6F, G) and ROS production (Fig. 6H, I) were also promoted by deficiency of NMDAζ1, NMDAε2, and NMDAε4. The mitochondrial function was also determined in NMDAR-deficient HUVECs. Like the above results, the mitochondrial function, including OCR (Fig. 6J), basal respiration rate (Fig. 6K), maximal respiration rate (Fig. 6L), and ATP production (Fig. 6M) was significantly restrained when knockdown of NMDAζ1, NMDAε2, and NMDAε4, but not NMDAε1 and NMDAε3. These results strongly suggested that decreasing the three subunits of NMDA receptors, NMDAζ1, NMDAε2, and NMDAε4, promotes cell apoptosis by promoting Ca^2+^ uptake, ROS production, and mitochondrial dysfunction.

### MK801 alleviated impairment of vascular permeability in LPS-induced mice

**Fig. 7:**
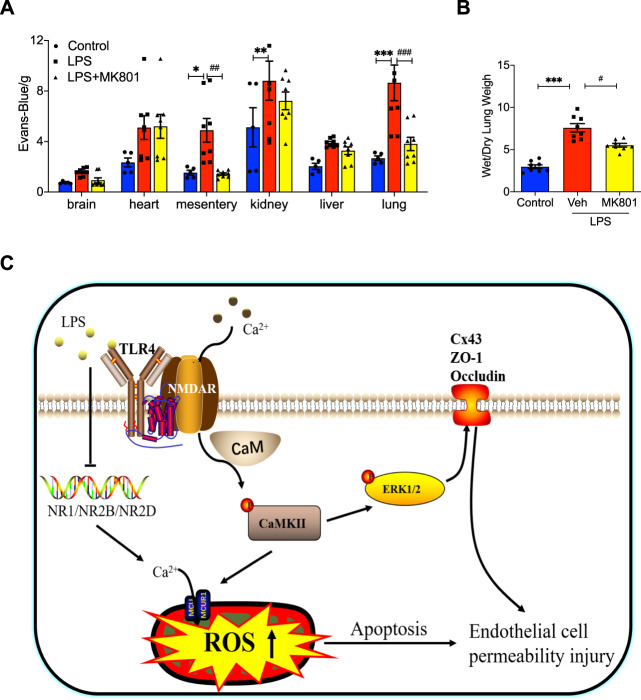
MK801 ameliorated LPS-induced vascular permeability in mice. **A** EB extravasation in C57/BL6 mice treated with vehicle, LPS (5 mg/kg, i/p), or LPS plus MK-801 for 12 h. Evans blue dye (30 ml/kg, i/v) was injected 2 h before the termination of the experiment. **B** Wet/dry ratio of lungs from the control group, LPS-induced mice group, and LPS-induced mice treated with MK-801. **C** The schematic image of the present finding. (Data are presented as mean ± SEM, ***P < 0.001, **P < 0.01, *P < 0.05 vs. Control; ##*P* < 0.01, #*P* < 0.05 vs. LPS).

The In vitro results have demonstrated that MK801 can significantly suppress LPS-induced cell permeability in HUVECs. To further validate the protective effect of MK801 in LPS-induced injury in vivo, we used LPS to induce a systemic immune response in mice and treated them with MK801. The results exhibited that LPS-increased vascular permeability in mesentery, kidneys, liver, and lung. However, treatment with MK801 notably suppressed the vascular permeability injury in mesentery and lung (Fig. 7A). In addition, we also observed the ratio of wet/dry weight in the lung and found that the proportion of wet/dry weight was significantly increased in the lung of LPS-induced mice, which can be reversed by MK801. These results suggested that NMDAR inhibitor MK801 can protect mice from LPS-induced acute lung by inhibiting the impairment of vascular permeability.

## Discussion

Most patients underwent cardiovascular surgery have symptom of acute lung injury (ALI) symptom. It is characterized by pulmonary edema caused by diffuse alveolar‑capillary membrane injury and appears as respiratory distress refractory hypoxemia [25]. Acute respiratory distress syndrome (ARDS), a much tougher form of ALI, can quickly development into multiple organ failure and poor diagnosis among patients [26]. Although it has a significant inhibitory role on the inflammatory response during ALI, clinical trials have shown that hormonal drugs cause unexpected adverse side roles in clinical use [27]. Previous epidemiological findings have revealed that LPS, a critical component of the cell wall in gram‑negative bacteria, is the most common cause of ALI and has been widely used to establish animal models of ALI [28, 29]. LPS can activates NF‑κB by binding to leukocyte TLR4, causing the expression of inflammatory factors, such as TNF‑α, IL‑1β, and IL‑6, leading to paracellular junctions disruption [30, 31]. Although the application of a lung-protective ventilation strategy has reduced the mortality of ALI, there is currently no clinically effective drug to treat ALI effectively. Thus, profoundly understanding the pathology of ALI and seeking new therapeutic targets are of great importance.

NMDARs are highly permeable to Ca^2+^ influx and essential for cell biology [32]. A previous study has shown that NMDARs overactivation induced Ca^2+^ overload inside the neurons, leading to neuronal death following the damage [33]. Our present study observed that the NMDAR antagonist MK801 protected against LPS-induced cell death and prevented intracellular Ca^2+^ and glutamic acid release. An increasing body of evidence shows that Ca^2+^ signaling is critical for the LPS-triggered immune response. Wong et al. reported that LPS induces inflammation by activation of the store-operated calcium (SOC) channel and NF-κB/ERK1/2 pathway in gastric cancer cells [34]; SOC channel was also found to participate in LPS-treated astrocyte activation [35]; in macrophage, transient receptor potential melastatin-like 7 (TRPM7), a non-selective but Ca^2+^-conducting ion channel, regulates LPS-induced macrophage activation by promoting cytosolic Ca^2+^ elevations [36]. Our further finding exhibited that MK801 decreased LPS-induced Ca^2+^ uptake, mainly via regulating the phosphorylation of CAMKII and ERK pathways. The finding that CAMKII is involved in LPS-induced insult is similar to two previous studies in macrophages, which reported that CAMK [37] or CAMKIV [38] play a significant role in LPS-induced cell response, indicating CAMKs are crucial in LPS-induced cell dysfunction.

NMDARs, a family of glutamate-gated ion channels, play an important role in regulating synaptic function in various disorders [39]. NMDARs are heteromeric assemblies of NR1, NR2, and NR3 subunits, co-translationally assembled in the endoplasmic reticulum (ER) forming functional channels with differing physiological and pharmacological properties and distinct patterns of synaptic targeting [40, 41]. Each NMDAR subunit includes a big amino (N)-terminal extracellular region, three membrane-spanning region, a re-entry, or ‘hairpin’ loop forming the pore-lining region (membrane domain 2), and an intracellular carboxyl (C)-terminal region [32]. Our present study found that utilizing NAMDARs antagonist MK801 significantly decreased LPS-induced mitochondria dysfunction, such as ROS production, oxygen consumption, and ATP production in mitochondria. Moreover, we found TRL4 was specifically immunoprecipitated with the three subtypes of NMDARs, such as NMDARs, NMDAζ1, NMDAε2, and NMDAε4, but not NMDAε1 and NMDAε3, suggesting that NMDAζ1/NMDAε2/NMDAε4 complex dominantly interacted with TRL4. Interestingly, further investigation in the present study observed that MK801 exerts its function in LPS-induced cell injury by promoting LPS-decreased expression of NMDAζ1, NMDAε2, and NMDAε4, indicating that restrained expression of NMDAζ1, NMDAε2, and NMDAε4 can significantly result in the HUVECs damage. The effect of MK801 on the expression of NMDARs was consistent with previous literature that reported administration of MK801 can promote the expression of NMDARs in young adult rat prefrontal cortex in vivo [24]. Further observation has validated that the integrity of NMDARs is vital for cell function, in which deficiency of NMDAζ1 and NMDAε4 resulted in cell apoptosis, Ca^2+^ overload, ROS production, and mitochondria dysfunction.

Pulmonary vascular endothelial damage induced by LPS results in the occurrence and further progression of ALI/ARDS [42]. A LPS-induced approach has been extensively used to generate an ALI animal model by triggering inflammatory responses, especially in vascular endothelial cells [43, 44]. In our present study, the LPS-induced ALI animal model was employed. The in vivo study has demonstrated that the NMDAR inhibitor MK801 can long-lastingly block the activity of NMDARs and exhibit a severe protective role by promoting permeability in the lung and heart. This finding certified the protective effect of MK801 in LPS-treated HUVECs and is the most exciting finding in our present study.

As shown in the schematic in Fig. 7C, the present study provides a novel mechanism by which NMDARs regulate LPS-induced cell dysfunction. Specifically, inhibiting NMDARs by MK801 can significantly decrease LPS-induced cell death, mainly by regulating intra/extracellular glutamate, cellular Ca^2+^ influx, and ROS production via CAMKII/Cam/Erk pathway. Inhibition of NMDARs in LPS-treated HUVECs markedly decreased ROS production. It ameliorated mitochondrial function by restrained membrane potential of mitochondria, mitochondria Ca^2+^ uptake, and improved oxygen consumption in mitochondria, which is mainly regulated by oxidative phosphorylation-related protein SDH2, MTCO1, and ATP5A via CaM/CAMKII pathways. MK801 facilitated LPS-induced cell permeability by regulating the expression of ZO-1, Occludin, and Cx43 via the Erk pathway in vitro. Interestingly, in vivo effect of MK801 on LPS-induced impairment of vascular permeability in mice has also been validated. The co-immunoprecipitated results suggested that three subtypes of NMDARs, NMDAζ1, NMDAε2, and NMDAε4, can specifically interact with TLR4. Most interestingly, MK801 can reverse LPS-decreased expression of NMDAζ1, NMDAε2, and NMDAε4, which was also confirmed in the results of knockdown of NMDAζ1, NMDAε2, and NMDAε4. Our finding may provide a novel therapeutic target for acute lung injury by inhibiting NMDARs.

However, there are some controversial aspects of this study. The NMDAR inhibitor MK801 has a protective effect on brain, kidney, and liver, but MK801 may also cause neurological damage at high doses. This study demonstrated that MK801 (10 mg/kg) significantly attenuated LPS-induced increases in mesenteric and pulmonary permeability without significant improvement in other tissues, which does not exclude the existence of neurotoxicity of MK801 at this dose. However, the results still support that MK801 may protect against acute lung injury in mice by inhibiting vascular permeability damage. As for the dose-dependent protective effect of MK801 in in vivo experiments and the critical dose for possible toxicity to other organs, we will further explore this in depth.

## Materials and methods

### Cell culture and viability assay

Human umbilical vein endothelial cells (HUVECs) were obtained from ScienCell Research Laboratories (San Diego, CA, USA) and cultured in plates pre-coated with 0.2% gelatin in endothelial cell medium (ECM, ScienCell, San Diego, CA, USA). For cell viability assay, cells were seeded in 96-well plates and grew to 70–80% confluence, then exposed to LPS (20 μg/ml) in the absence or presence of MK801 at desired concentrations (1, 5, 10 μM) for 24 h. Cell viability was determined by 3-(4,5-dimethyl-2-thiazolyl)-2,5-diphenyl-2-H-tetrazolium bromide (MTT, Solarbio Technology, Beijing, China) assay as described previously [45, 46]. After treatment, cells were incubated with 0.5 mg/mL MTT for 4 h and resuspended in 150 μL of DMSO (Sigma-Aldrich, USA). Absorbance was measured at 495 nm using an Infinite M200 Pro Nano quant (TECAN, Switzerland).

### Measurement of endothelial permeability

Endothelial monolayer permeability was determined by an In Vitro Vascular Permeability Assay (24-well) (Sigma-Aldrich, USA) based on a Transwell model (Millipore, USA). Briefly, HUVECs (2 × 10^5^/well) were seeded into the Transwell culture plates. The inserts were placed into 24-well plates containing 500 μL 5% ECM, and the cells were allowed to grow to full confluence until a monolayer formed. After treatment, 200 μL FITC-dextran (2 mg/mL) was added to the inserts and allowed to pass through the cell monolayer for 4 h. Subsequently, a 50 μL culture medium from the low chamber was harvested to determine the fluorescence intensity on a fluorescent microplate reader at excitation and emission wavelengths of 485 nm and 530 nm, respectively.

### Immunofluorescence for F-actin analysis

Phalloidin-TRITC (Solarbio Technology, Beijing, China) was applied to determine the cytoskeleton by immunofluorescence staining with F-actin. Briefly, HUVECs were plated on confocal culture dishes and treated with LPS in the presence or absence of different concentrations of MK801 for 24 h. Cells were then washed with PBS 3-times and fixed in 4% paraformaldehyde for 10 min. After permeabilization with 0.5% Triton X-100 (Sigma-Aldrich) for 5 min, followed by a 30 min incubation with Phalloidin-TRITC at 37 °C, cells were mounted on DAPI-containing mounting media (Solarbio Technology). Images were captured with a fluorescent microscope (EVOS™ FL Auto 2 Imaging System, Thermo Fisher, USA).

### Flow cytometry analysis

Flow cytometry analysis was employed to determine cell apoptosis, intracellular calcium level, ROS production, mitochondrial calcium level, and mitochondrial transmembrane potential in HUVECs using a flow cytometer (Beckman Coulter, USA) as previously described [47, 48]. The detailed methods of flow cytometry analyses were shown in the Supplemental information.

### Glutamic acid contents determination

The intracellular and extracellular glutamic acid concentrations were determined in harvested HUVECs or supernatant medium. After treatment, cells were harvested, rinsed with PBS, and centrifuged at 4000 rpm for 10 min. Glutamic acid contents of harvested cells or supernatant medium were measured using a Micro Glutamic Acid Content Assay Kit (BC1585, Solarbio Technology, China) following the manufacturer’s instructions. Contents were determined by comparing the absorbance value with the calibration plot for standard solutions.

### Dynamic evaluation of mitochondrial calcium

The dynamic calcium in mitochondria was evaluated with a mitochondrial-specific indicator Rhod-2 using a Laser Scanning Confocal Microscope (Leica TCS SP5II, Germany). To assess the effect of LPS and/or MK801 on mitochondrial calcium uptake, a mitochondrial calcium uniporter (MCU) inhibitor DS16570511(MCE, NJ, USA) and a mitochondrial calcium uptake inhibitor Ru360 (MCE, NJ, USA) were applied to investigate the changes in the mitochondrial calcium level. HUVECs were pre-incubated with MK801 5 μM, DS1657051 10 μM, or Ru360 10 μM for 2 h. After incubation of Rhod-2 AM for 30 min, cells were exposed to LPS to measure mitochondrial dynamic calcium levels. The fluorescence was continuously monitored at 5 s intervals for up to 7200 s using a Laser Scanning Confocal Microscope at an excitation wavelength of 549 nm and an emission wavelength of 578 nm. The continuous changes in the calcium-dependent fluorescence for each trace were calculated using relative fluorescence units (RFUs).

### Measurement of mitochondrial oxygen consumption

The oxygen consumption rate was measured in HUVECs at 37 °C in a Seahorse XF24 Extracellular Flux Analyzer (Seahorse Bioscience, USA). According to the manufacturer’s instructions, preliminary experiments were performed to select optimal seeding density (2 × 10^5^ cells/well) and compound concentrations. After being exposed to LPS (20 μg/ml) for 24 h in the absence or presence of MK801 (1, 5, 10 μM), or transfected with different subtypes of NMDARs for 48 h, cells were sequentially exposed to oligomycin, FCCP, and rotenone, using the XF Cell Mito Stress Kit (Seahorse Bioscience, MA, USA).

### Small interfering RNA (siRNA)

The siRNA technique was used to silence subtypes of NMDARs in HUVECs. Cells at a 40–50% confluence were transfected with specific siRNA duplexes (Santa Cruz Biotechnology, CA, USA) using a Transfection Reagent (Zeta Life, San Francisco, USA) following the manufacturer’s instructions. After transfection of control siRNA (sc-37007), NMDAζ1 siRNA (sc-36081), NMDAε1 siRNA (sc-36083), NMDAε2 siRNA (sc-36085), NMDAε3 siRNA (sc-42546), NMDAε4 siRNA (sc-36087), or TLR4 siRNA (sc-40260) (Santa Cruz Biotechnology, Dallas, TX, USA) for 48 h, cells were then collected for western blot, flow cytometry, and mitochondrial functional analysis.

### Co-immunoprecipitation and western blot analysis

Co-immunoprecipitation (Co-IP) and western blot analysis were employed to determine the combination and expression of specific proteins in cultured HUVECs. For the Co-IP assay, briefly, cells were harvested with RIPA lysis buffer (Catalog #AR0105, Boster Biological Technology, CA, USA) with protease and phosphatase inhibitors. Supernatants were collected, and protein concentration was determined using a BCA protein assay kit (Solarbio, Beijing, China). Each immunoprecipitation (IP) was carried out using a 10 μg antibody and 500 μg protein. The precipitated proteins were collected using protein A + G beads (Santa Cruz Biotechnology, Dallas, TX, USA), washed, eluted in boiling Laemmli sample buffer, and subjected to Western blot. For western blot, the Co-IP samples or directly lysis buffer harvested samples were separated with 10–15% SDS-polyacrylamide gels and transferred to PVDF membranes (Bio-Rad, Hercules, CA, USA). The membranes were blocked with 5% non-fat milk for 1 h and then incubated with primary antibodies (1:1000) at 4 °C overnight. After washout, membranes were incubated with HRP-conjugated secondary antibodies (1:10,000) for 1 h at room temperature. Blots were visualized with ECL-TM reagents (Advansta, Menlo Park, CA, USA). The protein signals were captured with a Fluor Chem E chemiluminescence detection system (Bio-Rad, Hercules, CA, USA). All cellular Western blots were repeated at least five times. The signal intensity of the immunoreactive bands was quantified using Image J software (NIH, Bethesda, MD, United States) and normalized to that of β-actin in each sample. The anti-pErk (#4370S) and anti-Erk (#4695S) primary antibodies were from Cell Signaling Technology (Danvers, MA, USA); anti-β-actin (sc-47778), anti-CaM (sc-137079), anti-TLR4 (sc-293072), anti-ZO-1 (sc-33725), anti-Cx43 (sc-271837), anti-Occludin (sc-133256), anti-NMDAζ1 (sc-518053), anti-NMDAε1 (sc-515148), anti-NMDAε2 (sc-365597), and anti-NMDAε4 (sc-17822) primary antibodies were from Santa Cruz; anti-MCUR1 (A08547-1) was from BOSTER Technology (Wuhan, China); anti-pCaMKII (ab124880), anti-CaMKII (ab52476), anti-MCU (ab219827), anti-NDUFB8 (ab192878), anti-Uqcrc2 (ab203832), anti-SDHB (ab175225), anti-MTCO1 (ab203912), and anti-ATP5A (ab176569) antibodies were from Abcam (Cambridge, UK).

### Animal experiments

The animal experiments were approved by the Ethics Committee of Xiamen University (XMU-LAC20190120). Male C57BL/6 mice were obtained from Beijing Vital River Laboratory Animal Technology (Beijing, China) and raised in the Laboratory Animal Center of Xiamen University. Mice (20–25 g) aged 6 to 8 weeks were maintained at room temperature (23 ± 2 °C) with a 12 h light/dark cycle and free access to essential diet and water. The mice were randomly assigned to the following three groups: (1) control group (*n* = 5); (2) LPS-induced group (*n* = 8); and (3) LPS-induced and MK801-treated group (*n* = 8). After 1 week of adaptation to laboratory conditions, LPS (5 mg/kg body weight; Sigma-Aldrich Technology, Gillingham, USA), MK801 (10 mg/kg body weight), or sterile saline were administered intraperitoneal injection (i.p.). Mice were injected between 8:00 and 9:00 AM to avoid diurnal variation in response to LPS, maintained in a heated recovery cage, monitored for adverse effects, and given a subcutaneous saline injection following local ethical requirements. After 24-h injection, animals were injected with Evans blue dye (EBD, dissolved in 0.9% saline) for blood vessel permeability assessment, anesthetized with 4% followed by 2% isoflurane, and sacrificed. The lung, liver, heart, brain, mesentery, and kidneys were harvested for analysis. For assessment of LPS-induced lung edema, the right lung tissues were excised. The surface blood and water on the lung were absorbed by filter paper. The wet weight (W) was immediately measured, and then the tissue was placed in an oven at 70 °C for 72 h to obtain the dry weight (D). The ratio of the wet lung to the dry lung was calculated to assess lung edema.

### Blood vessel permeability measurement

The EBD was injected at a dose of 20 mg/kg body weight 30 min before termination of the experiment via tail vein. After euthanasia with isoflurane, animals were subjected to perfusion through the right ventricle with EBD. The organs were harvested and weighed, homogenized in PBS by mechanical mincing using a tissue homogenizer, and incubated with formamide (vol/vol 2:1 ratio). The mixture was incubated at 60 °C for 24 h. After incubation, the tissue homogenate was centrifuged at 5000 rpm for 30 min. The absorbance of EBD in the tissue supernatants was performed with a spectrophotometer at 620 and 740 nm.

### Statistical analysis

Statistical analyses were performed using GraphPad Prism 9.0 software (GraphPad Software, Inc., San Diego, CA, USA). All data are presented as the mean ± SEM. The investigators were not blinded to the group allocation during the study. The data were normalized using the Shapiro-Wilk normality test. *F*-test for two groups and Bartlett’s test for ≥3 groups were used for equal variance. To compare data without equal variance, the Mann–Whitney test was used to compare 2 groups, and the Kruskal–Wallis test was used to compare ≥ 3 groups. Other data were analyzed by the Student t-test and a one-way ANOVA. Statistical analyses of the chemotaxis assay were performed by a two-way ANOVA. For post hoc analysis, the Sidak, Tukey, or Dunnett test was performed to correct multiple comparisons, and the Fisher Least Significant Difference test was used for planned comparisons. The figures or figure legends show the sample size and statistical tests. *P* values < 0.05 were considered statistically significant.

## Supplementary information

Supplemental material

## Data Availability

All data generated or analyzed during this study are included in this published article and its supplementary information files.
