# Supplementary material for: NMDARs antagonist MK801 suppresses LPS-induced apoptosis and mitochondrial dysfunction by regulating subunits of NMDARs via the CaM/CaMKII/ERK pathway
Source: Cell Death Discov. 2023 Feb 11;9:59. doi: 10.1038/s41420-023-01362-9 (PMC9922289; doi:10.1038/s41420-023-01362-9)
Supplement: Supplementary file 1 — Supplemental material [file 41420_2023_1362_MOESM1_ESM.docx]

**Supplemental material**

**1. Supplemental methods**

**Flow cytometry analysis**

The flow cytometry analysis was employed to assay the viability and apoptosis, intracellular calcium level, ROS production, mitochondrial calcium level, and mitochondrial transmembrane potential in HUVECs using a flow cytometer (Beckman Coulter, United States). For viability and apoptosis determination, annexin V and propidium iodide staining were performed with the Annexin V-FITC apoptosis detection kit (Dojindo Molecular Technologies, Rockville, MD, United States) following the manufacturer’s instruction, and the stained cells were subjected to flow cytometry within 1 h. For ROS production analysis, the cells exposed to LPS in the absence and presence of MK-801 were incubated with 10 μM DCFH-DA (Thermo Fisher Scientific, Waltham, MA, United States) in fresh ECM at 37°C for 30 min, and then intracellular ROS level was determined.

To determine mitochondrial transmembrane potential, HUVECs with different treatments were incubated with 2 μM JC-1 (Solarbio Technology, Beijing, China) at 37°C for 30 min. Green fluorescence reflected the monomeric form of JC-1, and red fluorescence reflected the aggregate form. Mitochondrial membrane potential was then analyzed with a Flow cytometer.

For intracellular and mitochondrial calcium determination, after incubation of LPS in the absence and presence of MK-801, HUVECs were treated with 1 μM Fluo-4 AM (Thermo Fisher Scientific, Waltham, MA, United States) and 5 μM Rhod-2 AM (Thermo Fisher Scientific, Waltham, MA, United States) at 37°C for 30 min, and then intra-cellular and mitochondrial calcium were determined.

To evaluate the binding of LPS to HUVECs, cells were incubated with LPS-FITC（20 µg/ml; Sigma-Aldrich, UK) for 24 h in the absence or presence of MK-801 at 37°C, 5% CO_2_. After incubation, cells were washed with PBS and analyzed with a Flow cytometer.

**2. Original western blotting images**

**Figure 2H**

19KD

NDUFB8

Lane 1: Control; 2: LPS; 3: LPS+1 uM MK801; 4: LPS+5 uM MK801; 5: LPS+10 uM MK801; 6: none; 7: Control; 8: LPS; 9: LPS+1 uM MK801; 10: LPS+5 uM MK801; 11: LPS+10 uM MK801

32KD

SDHB

Lane 1: Control; 2: LPS; 3: LPS+1 uM MK801; 4: LPS+5 uM MK801; 5: LPS+10 uM MK801; 6: none; 7: Control; 8: LPS; 9: LPS+1 uM MK801; 10: LPS+5 uM MK801; 11: LPS+10 uM MK801

Uqcrc2

48KD

Lane 1: Control; 2: LPS; 3: LPS+1 uM MK801; 4: LPS+5 uM MK801; 5: LPS+10 uM MK801

57KD

MTCO1

Lane 1: Control; 2: LPS; 3: LPS+1 uM MK801; 4: LPS+5 uM MK801; 5: LPS+10 uM MK801; 6: none; 7: Control; 8: LPS; 9: LPS+1 uM MK801; 10: LPS+5 uM MK801; 11: LPS+10 uM MK801

ATP5A

60KD

Lane 1: Control; 2: LPS; 3: LPS+1 uM MK801; 4: LPS+5 uM MK801; 5: LPS+10 uM MK801; 6: none; 7: Control; 8: LPS; 9: LPS+1 uM MK801; 10: LPS+5 uM MK801; 11: LPS+10 uM MK801

β-actin

43KD

Lane 1: Control; 2: LPS; 3: LPS+1 uM MK801; 4: LPS+5 uM MK801; 5: LPS+10 uM MK801; 6: none; 7: Control; 8: LPS; 9: LPS+1 uM MK801; 10: LPS+5 uM MK801; 11: LPS+10 uM MK801

**Figure 3A**

CaM

17KD

Lane 1: Control; 2: LPS; 3: LPS+BAPTA; 4: LPS+W-7; 5: none; 6: none; 7: Control; 8: LPS; 9: LPS+BAPTA; 10: LPS+W-7

p-Erk

42/44KD

Lane 1: Control; 2: LPS; 3: LPS+BAPTA; 4: LPS+W-7; 5: none; 6: none; 7: Control; 8: LPS; 9: LPS+BAPTA; 10: LPS+W-7

t-Erk

42/44KD

Lane 1: Control; 2: LPS; 3: LPS+BAPTA; 4: LPS+W-7; 5: none; 6: none; 7: Control; 8: LPS; 9: LPS+BAPTA; 10: LPS+W-7

p-CaMKII

56KD

Lane 1: Control; 2: LPS; 3: LPS+BAPTA; 4: LPS+W-7; 5: none; 6: none; 7: Control; 8: LPS; 9: LPS+BAPTA; 10: LPS+W-7

t-CaMKII

56KD

Lane 1: Control; 2: LPS; 3: LPS+BAPTA; 4: LPS+W-7; 5: none; 6: none; 7: Control; 8: LPS; 9: LPS+BAPTA; 10: LPS+W-7

β-actin

43KD

Lane 1: Control; 2: LPS; 3: LPS+BAPTA; 4: LPS+W-7; 5: none; 6: none; 7: Control; 8: LPS; 9: LPS+BAPTA; 10: LPS+W-7

p-Erk

42/44KD

**Figure 3C**

Lane 1: Control; 2: LPS; 3: LPS+1 uM MK801; 4: LPS+5 uM MK801; 5: LPS+10 uM MK801

t-Erk

42/44KD

Lane 1: Control; 2: LPS; 3: LPS+1 uM MK801; 4: LPS+5 uM MK801; 5: LPS+10 uM MK801; 6: none; 7: Control; 8: LPS; 9: LPS+1 uM MK801; 10: LPS+5 uM MK801; 11: LPS+10 uM MK801

CaM

17KD

Lane 1: Control; 2: LPS; 3: LPS+1 uM MK801; 4: LPS+5 uM MK801; 5: LPS+10 uM MK801

MCUR1

40KD

Lane 1: Control; 2: LPS; 3: LPS+1 uM MK801; 4: LPS+5 uM MK801; 5: LPS+10 uM MK801; 6: none; 7: Control; 8: LPS; 9: LPS+1 uM MK801; 10: LPS+5 uM MK801; 11: LPS+10 uM MK801

MCU

35KD

Lane 1: Control; 2: LPS; 3: LPS+1 uM MK801; 4: LPS+5 uM MK801; 5: LPS+10 uM MK801

TLR4

120KD

Lane 1: Control; 2: LPS; 3: LPS+1 uM MK801; 4: LPS+5 uM MK801; 5: LPS+10 uM MK801

β-actin

43KD

Lane 1: Control; 2: LPS; 3: LPS+1 uM MK801; 4: LPS+5 uM MK801; 5: LPS+10 uM MK801

t-CaMKII

56KD

Lane 1: Control; 2: LPS; 3: LPS+1 uM MK801; 4: LPS+5 uM MK801; 5: LPS+10 uM MK801; 6: Control; 7: LPS; 8: LPS+1 uM MK801; 9: LPS+5 uM MK801; 10: LPS+10 uM MK801

56KD

p-CaMKII

Lane 1: Control; 2: LPS; 3: LPS+1 uM MK801; 4: LPS+5 uM MK801; 5: LPS+10 uM MK801; 6: Control; 7: LPS; 8: LPS+1 uM MK801; 9: LPS+5 uM MK801; 10: LPS+10 uM MK801

43KD

β-actin

Lane 1: Control; 2: LPS; 3: LPS+1 uM MK801; 4: LPS+5 uM MK801; 5: LPS+10 uM MK801; 6: Control; 7: LPS; 8: LPS+1 uM MK801; 9: LPS+5 uM MK801; 10: LPS+10 uM MK801

**Figure 3E**

t-CaMKII

56KD

Lane 1: Marker; 2: control; 3: LPS; 4: LPS+KN93; 5: Marker; 6: control; 7: LPS; 8: LPS+KN93; 9: Marker; 10: control; 11: LPS; 12: LPS+KN93; 13: Marker; 14: Marker

p-CaMKII

56KD

Lane 1: Marker; 2: control; 3: LPS; 4: LPS+KN93; 5: Marker; 6: control; 7: LPS; 8: LPS+KN93; 9: Marker; 10: control; 11: LPS; 12:LPS+KN93; 13: Marker

p-Erk

42/44KD

Lane 1: control; 2: LPS; 3: LPS+KN93; 4: Marker; 5: control; 6: LPS; 7: LPS+KN93; 8: Marker; 9: control; 10: LPS; 11: LPS+KN93

t-Erk

42/44KD

Lane 1: control; 2: LPS; 3: LPS+KN93; 4: Marker; 5: control; 6: LPS; 7: LPS+KN93; 8: Marker; 9: control; 10: LPS; 11: LPS+KN93

MCUR1

40KD

Lane: 1: Marker; 2: control; 3: LPS; 4: LPS+KN93; 5: Marker; 6: control; 7: LPS; 8:LPS+KN93; 9: Marker; 10:control; 11:LPS; 12:LPS+KN93; 13: Marker; 14: Marker

MCU

35KD

Lane: 1: Marker; 2: control; 3: LPS; 4: LPS+KN93; 5: Marker; 6: control; 7:LPS; 8:LPS+KN93; 9: Marker; 10:control; 11:LPS; 12:LPS+KN93; 13: Marker; 14: Marker

MTCO1

57KD

Lane 1: Marker; 2: control; 3: LPS; 4:LPS+KN93; 5: Marker; 6: control; 7:LPS; 8:LPS+KN93; 9: Marker; 10:control; 11:LPS; 12:LPS+KN93; 13: Marker

SDHB2

32KD

Lane 1: Marker; 2: Marker; 3: control; 4: LPS; 5: LPS+KN93; 6: Marker; 7: control; 8: LPS; 9: LPS+KN93; 10: Marker; 11: control; 12: LPS; 13: LPS+KN93; 14: Marker

ATP5a

60KD

Lane 1: Marker; 2: Marker; 3: control; 4: LPS; 5: LPS+KN93; 6: Marker; 7: control; 8: LPS; 9: LPS+KN93; 10: Marker; 11: control; 12: LPS; 13: LPS+KN93; 14: Marker

β-actin

43KD

Lane 1: Marker; 2: Marker; 3: control; 4: LPS; 5: LPS+KN93; 6: Marker; 7: control; 8: LPS; 9: LPS+KN93; 10: Marker; 11: control; 12: LPS; 13: LPS+KN93; 14: Marker

**Figure 4D**

ZO-1

220KD

Occludin

60KD

Lane: 1: Marker; 2: Marker; 3: Control; 4: LPS; 5: LPS+1 uM MK801; 6: LPS+5 uM MK801; 7: LPS+10 uM MK801; 8: Control; 9: LPS; 10: LPS+1 uM MK801; 11: LPS+5 uM MK801; 12: LPS+10 uM MK801; 13: Marker; 14: Marker

Cx43

43KD

Lane 1: Control; 2: LPS; 3: LPS+1 uM MK801; 4: LPS+5 uM MK801; 5: LPS+10 uM MK801; 6: Control; 7: LPS; 8: LPS+1 uM MK801; 9: LPS+5 uM MK801; 10: LPS+10 uM MK801

β-actin

43KD

Lane 1: Control; 2: LPS; 3: LPS+1 uM MK801; 4: LPS+5 uM MK801; 5: LPS+10 uM MK801; 6: Control; 7: LPS; 8: LPS+1 uM MK801; 9: LPS+5 uM MK801; 10: LPS+10 uM MK801

**Figure 4F**

Occludin

60KD

Lane 1: Marker; 2: control; 3: LPS; 4: LPS+PD98; 5: Marker; 6: control; 7: LPS; 8: LPS+PD98; 9: Marker; 10: control; 11: LPS; 12: LPS+PD98; 13: Marker

Cx43

43KD

Lane 1: Marker; 2: Marker; 3: control; 4: LPS; 5: LPS+PD98; 6: Marker; 7: control; 8: LPS; 9: LPS+PD98; 10: Marker; 11: control; 12: LPS; 13: LPS+PD98; 14: Marker; 15: Marker

ZO-1

220KD

Lane 1: Marker; 2: control; 3: LPS; 4: LPS+PD98; 5: Marker; 6: control; 7: LPS; 8: LPS+PD98; 9: Marker; 10: control; 11: LPS; 12: LPS+PD98; 13: Marker

t-Erk

42/44KD

1: control; 2: LPS; 3: LPS+PD98; 4: Marker; 5: control; 6: LPS; 7: LPS+PD98; 8: Marker; 9: control; 10: LPS; 11: LPS+PD98

p-Erk

42/44KD

1: control; 2: LPS; 3: LPS+PD98; 4: Marker; 5: control; 6: LPS; 7: LPS+PD98; 8: Marker; 9: control; 10: LPS; 11: LPS+PD98

β-actin

43KD

Lane 1: Marker; 2: Marker; 3: control; 4: LPS; 5: LPS+PD98; 6: Marker; 7: control; 8: LPS; 9: LPS+PD98; 10: Marker; 11: control; 12: LPS; 13: LPS+PD98; 14: Marker; 15: Marker

**Figure 6C**

IB: NMDAζ1

105/120 KD

Lane 1: Marker; 2: Marker; 3: Control+anti-TLR4; 4: LPS+anti-TLR4; 5: Control+IgG; 6: LPS+IgG; 7: Marker

177 KD

IB: NMDAε1

Lane 1: Control+anti-TLR4; 2: LPS+anti-TLR4; 3: Control+IgG; 4: LPS+IgG

IB: NMDAε2

178KD

Lane 1: Marker**;** 2: Control+anti-TLR4; 3: LPS+anti-TLR4; 4: Control+IgG; 5: LPS+IgG; 6: Marker

IB: NMDAε3

140 KD

Lane 1: Marker**;** 2: Control+anti-TLR4; 3: LPS+anti-TLR4; 4: Control+IgG; 5: LPS+IgG; 6: Marker

IB: NMDAε4

168 KD

Lane 1: Marker**;** 2: Marker**;** 3: Control+anti-TLR4; 4: LPS+anti-TLR4; 5: Control+IgG; 6: LPS+IgG; 7: Marker**;** 8: Marker

IB: TLR4

120 KD

Lane 1: Control+anti-TLR4; 2: LPS+anti-TLR4; 3: Control+IgG; 4: LPS+IgG

Input:

Lane 1: Marker**;** 2: Control+anti-TLR4; 3: LPS+anti-TLR4; 4: Control+IgG; 5: LPS+IgG; 6: Marker; 7: Marker

Input: NMDAε1

Lane 1: Marker**;** 2: Control+anti-TLR4 3: LPS+anti-TLR4; 4: Control+IgG; 5: LPS+IgG; 6: Marker; 7: Marker

IB: NMDAε2

Lane 1: Marker**;** 2: Control+anti-TLR4 3: LPS+anti-TLR4; 4: Control+IgG; 5: LPS+IgG; 6: Marker; 7: Marker

IB: NMDAε3

Lane 1: Marker**;** 2: Control+anti-TLR4 3: LPS+anti-TLR4; 4: Control+IgG; 5: LPS+IgG; 6: Marker; 7: Marker

Input: NMDAε4

Lane 1: Marker**;** 2: Control+anti-TLR4 3: LPS+anti-TLR4; 4: Control+IgG; 5: LPS+IgG; 6: Marker; 7: Marker

Input: TLR4

Lane 1: Marker**;** 2: Control+anti-TLR4 3: LPS+anti-TLR4; 4: Control+IgG; 5: LPS+IgG; 6: Marker; 7: Marker

**Figure 6D**

NMDAζ1

Lane: 1: Control; 2: Control; 3: Control; 4: LPS; 5: LPS; 6: LPS; 7: LPS+5 uM MK801; 8: LPS+5 uM MK801; 9: LPS+5 uM MK801; 10: LPS+10 uM MK801; 11: LPS+10 uM MK801; 12: LPS+10 uM MK801

NMDAε1

β-actin

Lane: 1: Control; 2: Control; 3: Control; 4: LPS; 5: LPS; 6: LPS; 7: LPS+5 uM MK801; 8: LPS+5 uM MK801; 9: LPS+5 uM MK801; 10: LPS+10 uM MK801; 11: LPS+10 uM MK801; 12: LPS+10 uM MK801

NMDAε4

Lane 1: Marker; 2: Control; 3: Control; 4: Control; 5: LPS; 6: LPS; 7: LPS; 8: LPS+5 uM MK801; 9: LPS+5 uM MK801; 10: LPS+5 uM MK801; 11: LPS+10 uM MK801; 12: LPS+10 uM MK801; 13: LPS+10 uM MK801; 14: Marker

β-actin

Lane: 1: Control; 2: Control; 3: Control; 4: LPS; 5: LPS; 6: LPS; 7: LPS+5 uM MK801; 8: LPS+5 uM MK801; 9: LPS+5 uM MK801; 10: LPS+10 uM MK801; 11: LPS+10 uM MK801; 12: LPS+10 uM MK801

NMDAε2

Lane 1: Control; 2: Control; 3: LPS; 4: LPS; 5: LPS+1 uM MK801; 6: LPS+1 uM MK801; 7: LPS+5 uM MK801; 8: LPS+5 uM MK801; 9: LPS+10 uM MK801; 10: LPS+10 uM MK801

β-actin

Lane 1: Control; 2: Control; 3: LPS; 4: LPS; 5: LPS+1 uM MK801; 6: LPS+1 uM MK801; 7: LPS+5 uM MK801; 8: LPS+5 uM MK801; 9: LPS+10 uM MK801; 10: LPS+10 uM MK801
